# School-based promotion of cessation support: reach of proactive mailings and acceptability of treatment in smoking parents recruited into cessation support through primary schools

**DOI:** 10.1186/1471-2458-13-381

**Published:** 2013-04-23

**Authors:** Kathrin Schuck, Roy Otten, Marloes Kleinjan, Jonathan B Bricker, Rutger CME Engels

**Affiliations:** 1 Behavioural Science Institute, Radboud University Nijmegen, Montessorilaan 3, P.O. Box 9104, 6500, HE Nijmegen, The Netherlands; 2 Fred Hutchinson Cancer Research Center, 1100 Fairview Avenue, P.O. Box 19024, Seattle, WA 98109, USA; 3 Department of Psychology, University of Washington, Box 351525, Seattle, WA 98195, USA

## Abstract

**Background:**

Several forms of cessation support have been shown effective in increasing the chance of successful smoking cessation, but cessation support is still underutilized among smokers. Proactive outreach to target audiences may increase use of cessation support.

**Methods:**

The present study evaluated the efficiency of using study invitation letters distributed through primary schools in recruiting smoking parents into cessation support (quitline support or a self-help brochure). Use and evaluation of cessation support among smoking parents were examined.

**Results:**

Findings indicate that recruitment of smokers into cessation support remains challenging. Once recruited, cessation support was well received by smoking parents. Of smokers allocated to quitline support, 88% accepted at least one counselling call. The average number of calls taken was high (5.7 out of 7 calls). Of smokers allocated to receive self-help material, 84% read at least some parts of the brochure. Of the intention-to-treat population, 81% and 69% were satisfied with quitline support or self-help material, respectively. Smoking parents were significantly more positive about quitline support compared to self-help material (*p*<.001).

**Conclusions:**

Cessation support is well-received and well-used among smoking parents recruited through primary schools. Future studies need to examine factors that influence the response to offers of cessation support in samples of nonvolunteer smokers.

**Trial registration:**

The protocol for this study is registered with the Netherlands Trial Register NTR2707

## Background

Cigarette smoking constitutes a serious burden to health and economy [1]. Connecting smokers to effective cessation services is a public health priority. The majority of smokers intend to quit smoking and a substantial proportion of smokers make repeated quit attempts [2]. When attempting to quit, relapse is the most probable outcome. Approximately, three-quarters of unaided quitters resume smoking within three months [3]. In a meta-analytic review of unaided smoking cessation, it was concluded that only 7% of unaided quit attempts last longer than 10 months [4]. Several forms of cessation support have been shown effective in increasing the chance of successful smoking cessation [5]. However, only a minority of smokers make use of such programs. In the United States, 37% of smokers who have tried to quit smoking report that they had ever read written material on smoking cessation, 12% had called a quitline, and 9% had attended individual counselling [6]. Similar rates on the use of cessation treatments are reported by Shiffman and colleagues [2]. In the Netherlands, one third of quitters report that they received assistance in quitting and less than 1% of smokers contact the national quitline [7].

Smoking parents represent an important subpopulation among adult smokers. Forty percent of smokers live with a child younger than 18 years old [8]. Twenty percent of parents are self-reported smokers [9]. Parental smoking is detrimental, not only to the parent, but also the child. A recent meta-analysis concluded that the risk of smoking uptake in adolescence is nearly threefold when both parents smoke [10]. Moreover, smoking parents frequently expose their children to second-hand smoke [11,12], which is associated with a variety of adverse health outcomes including childhood asthma, respiratory infections, and decreased lung growth in children [13,14]. Smoking parents may be particularly motivated to quit smoking. Smoker’s primary reasons for wanting to quit are concerns about the health consequences of their smoking [15]. Nearly two-thirds of adult smokers express concern for modelling smoking to children [8]. In a telephone survey, 64% of parent smokers indicated that they would accept telephone cessation support if recommended [9]. Also, parents of children with smoking-related illnesses display a particularly high motivation to quit [16,17]. Connecting smoking parents to cessation support may yield important health benefits for both parents and children. Parents who quit smoking will not only improve their own health, but will also reduce the risk of physical illness [18], smoking initiation [19], and regular smoking [20] in their children.

Proactive outreach may increase use of cessation support. Proactive outreach is the systematic targeting of all individuals in a defined population of smokers and the attempt to engage smokers with varying levels of motivation. Up to this point, efforts to engage smoking parents have almost exclusively focused on clinical settings [17,21,22]. While these efforts are valuable, proactive outreach of health care practices and hospitals may not extend to the general population of smoking parents. Public schools are a highly promising but understudied venue for reaching parents who smoke. Promoting cessation support through schools has the potential to reach a major proportion of smoking parents, thus yielding high potential public health impact. Also, schools are likely to constitute a ‘teachable setting’, that is, smokers may be more likely to make use of cessation support when reminded of their role as parents. To date, no study has evaluated the use of primary schools as a venue to promote smoking cessation among parents.

Previous studies have used varying approaches to increase smoker’s exposure to cessation support (e.g., direct mailings, health care provider outreach, telephone recruitment, or media advertisements). Offering cessation support through mailings has been shown to yield response rates between 2-11% in smokers identified from general practice and health care provider records [23-26]. Recruitment rates tend to be higher for interpersonal recruitment, with recruitment rates ranging between 44-65% [27-29]. While interpersonal recruitment (e.g., telephone recruitment) may constitute an efficient way to recruit smokers into clinical trials, this approach may be less feasible for implementation into the health care system, where few resources for recruitment are available. Though response rates vary considerably between studies and recruitment approaches, previous studies indicate that proactive outreach has considerable potential to connect smokers to cessation support.

Several forms of cessation support have demonstrated efficacy in increasing the chance of successful smoking cessation [5]. Telephone counselling, or quitline support, has been shown effective in increasing smoking cessation rates in a meta-analytic review [30]. Data from the European Smoking Cessation Helplines Evaluation study (ESCHER), which assesses cessation rates after quitline use in several European countries, showed point prevalent abstinence rates between 12% and 28% and prolonged abstinence rates between 4% and 15% at one-year follow-up [7]. Self-help materials (i.e., didactic materials giving information and advice on how to quit smoking) have also demonstrated efficacy in a meta-analytic review, which concluded that non-tailored self-help materials have a small benefit compared to no intervention [31]. Therefore, self-help materials constitute a cost-effective method to support otherwise unaided quit attempts, which can be disseminated easily and has the potential to help a large proportion of smokers.

The aim of the present study was two-fold: First, we sought to evaluate the reach of mailings distributed through primary schools in recruiting smoking parents into cessation support (i.e., school-based promotion of cessation support using mailings). Second, among smoking parents recruited into cessation support through primary schools, we compared use and acceptability of two cessation treatments with high potential public health impact: telephone counselling versus self-help material.

## Method

### Participants

Smoking parents were recruited through primary schools across several municipalities in the Netherlands. Primary schools were contacted by research assistants and were asked to distribute study invitation letters to parents. To increase the participation rate of schools, demands on schools were kept to a minimum (i.e., schools were asked to give the study invitation letters to the children and children were requested to give the letters to their parents). A total of 890 primary schools were contacted and 438 schools (49.2%) agreed to participate. In total, approximately 35,000 study invitation letters were mailed to schools. For the present study, schools were asked to give the letters only to children aged 9–12 years (Dutch grade 6–8; US grade 4–6). Study invitation letters included information about the study and eligibility criteria. Parents registered for the study by returning a form with their contact information in an enclosed envelope. Registration was also possible via e-mail, via telephone, or via the study website. Inclusion criteria were: 1) being at least a weekly smoker, 2) being a parent/caretaker of a child between 9–12 years old, 3) having the intention to quit smoking (currently or in the future), and 4) giving informed consent for participation of parent–child dyad. A total of 622 parents registered for the present study. A total of 512 parents were enrolled in the present study (returned informed consent form and baseline questionnaire).

### Procedure

**Figure 1:**
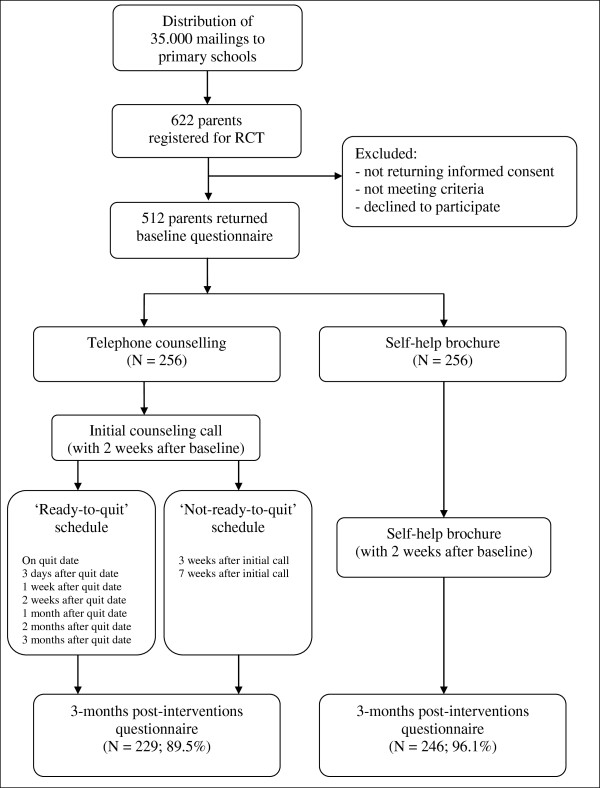
Flowchart.

An overview of the study design is presented in Figure 1. The baseline measurement took place between January and July 2011. Parents and children were asked to individually fill out a questionnaire (via a website or on paper). For the present study, only the parent data were used. More detailed information regarding the use of the child data can be found in the study protocol [32]. After the baseline assessment, parents were randomly assigned to either the telephone counselling condition (n=256) or the self-help brochure condition (n=256). A computer program was used to generate a randomization schedule. Allocation of participants to trial conditions was done by a member of the research group who was not involved in the present study. Participants were stratified by gender, educational level, and smoking intensity. Within 2 weeks after baseline assessment, parents were either called to schedule the first counselling call or they received the self-help brochure. The post-measurement took place approximately three months after start of the intervention (i.e., receiving the intake call or the self-help brochure). Further details on the study methodology can be found in the study protocol [32]. Parent–child couples received an incentive of 100 euro (approximately 127 US dollars) for their participation in all assessments. The ethics committee of the Faculty of Social Sciences at the Radboud University Nijmegen approved of the study.

### Conditions

#### Proactive telephone counselling

Participants in the telephone counselling condition received up to seven counsellor-initiated phone calls (i.e., one 30-minute intake call and up to six additional 10-minute calls) across a period of approximately three months. Telephone counselling was based on Motivational Interviewing [33] and cognitive-behavioural skill building. Counselling calls were conducted by counsellors of STIVORO, the non-profit Netherlands national quitline. All counsellors were trained and experienced in the delivery of telephone counselling.

During the intake call, the participants were asked if they wanted to set a quit date. Participants who wanted to set a quit date were encouraged to set a quit date within 10–12 days following the intake call. Subsequently, up to six additional phone calls were offered to support the initiation and maintenance of abstinence (Figure 1). Emphasis was put on psycho-education, intrinsic motivation for behavioural change, behavioural support, and relapse prevention. Participants who were not willing to set a quit a date were offered up to two additional phone calls (Figure 1). Emphasis was put on exploring ambivalence and increasing the participant’s intrinsic motivation to quit smoking using Motivational Interviewing [34]. If participants during any one call indicated that they wanted to set a quit date, they were offered additional phone calls to support the initiation and maintenance of abstinence.

In addition to the counselling calls, all participants in the telephone counselling condition received three accompanying booklets (4 pages, colour-print), which were designed specifically for the present study. Each booklet contained didactic information, tips and advice on how to initiate and maintain abstinence, motivational or self-efficacy enhancing messages, as well as ‘parent-relevant information’ (e.g., effects of SHS on children, suggestions to involve children in process of smoking cessation, strategies to manage parent-specific stressors). Participants received the booklets at three time points throughout telephone counselling (immediately after start of telephone counselling, three weeks after start of telephone counselling, and six weeks after start of telephone counselling).

#### Self-help brochure

Participants in the self-help condition received a 40-page, colour-printed self-help brochure ^a^ for smoking cessation copyrighted by Stivoro. The brochure included didactic information on nicotine dependence and the health benefits associated with quitting smoking, tips and advice on how to initiate and maintain abstinence, instruction in the use of cognitive and behavioural skills to avoid triggers to smoke and cope with urges to smoke, and strategies for managing a lapse or relapse to smoking. The brochure was divided into five parts: reasons for quitting, craving and withdrawal, preparing to quit, help with quitting, and maintenance of abstinence. The brochure was based on empirically supported practices for advice on smoking cessation, such as psycho-education, advice, tips, and exercises [31].

### Measures

#### Baseline characteristics

The baseline questionnaire included the variables gender, age, nationality, education, material status, employment status, cigarettes per day, years of smoking, nicotine dependence [FTND; [35], ever made a quit attempt and quit attempt in the past 12 months [36], intention to quit [8], other household smokers, and selected smoking-related illnesses of parent and child (e.g., cardiovascular disease, chronic respiratory illness).

#### Use and acceptability of cessation support

##### Telephone counselling condition

Participants in the telephone counselling condition were asked to report how many counselling calls they received (0, 1, 2, 3, 4, 5, 6, 7, 8 or more). Participants who received at least one counselling call were asked to which degree the call(s) helped with (1) motivation to quit or to stay quit, (2) coping with withdrawal symptoms, (3) coping with craving, (4) coping with situations that trigger craving, (5) prevention of a lapse or relapse, and (6) motivation to try again after a lapse or relapse. Ratings were: didn’t help, helped a little, and helped a lot. In addition, participants indicated to which degree they received emotional support and practical tips from the counsellors. Ratings were: not at all, a little, a lot. Also, participants were asked whether they had tried tips suggested during counselling (none, a few, a lot). Finally, participants indicated their satisfaction with the length of the intervention (too short, about right, too long), their overall satisfaction with telephone counselling (very unsatisfied, unsatisfied, satisfied, very satisfied), and whether they would make use of the STIVORO quitline again (no, yes).

Also, participants in the telephone counselling condition were asked how many accompanying booklets they received (0, 1, 2, 3). Recipients were asked to which extent they read the booklets (none or very little, less than half, more than half, in full) and whether they used tips provided in the booklets (none, a few, a lot). Also, recipients were asked to indicate to which extent the booklets helped with varying areas of difficulties and their overall satisfaction with the booklets (see above).

##### Self-help brochure condition

Participants in the self-help material condition were asked whether they received a brochure (yes, no). Recipients were asked to which extent they read the brochure (none or very little, less than half, more than half, in full) and whether they tried tips suggested in the brochure (none, a few, a lot). To evaluate acceptability of the brochure, recipients were asked the same questions about the brochure as participants in the telephone counselling condition were asked about the counselling calls (i.e., the extent the brochure helped with varying areas of difficulties, the extent to which participants received emotional support and practical tips, satisfaction with the length of brochure, overall satisfaction with brochure).

### Strategy for analysis

Participant characteristics at baseline are presented. To determine whether the randomization resulted in an equal baseline distribution of participant characteristics across conditions, chi-square tests and *t*-tests for independent samples were conducted. Use and acceptability of cessation support in both conditions are summarized. Differences between the two conditions in acceptability of cessation support were examined using chi-square tests. Post-measurement data are presented for recipients-only as well as for the intention-to-treat population. Statistical testing and report of results pertain to the intention-to-treat population.

### Attrition

At post-measurement, 229 participants (89.5%) completed the questionnaire in the telephone counselling condition and 246 (96.1%) completed the questionnaire in the self-help brochure condition. Attrition was significantly higher in the telephone counselling condition than the self-help brochure condition (*χ*^*2*^ = 8.42, *p*=.004). Participants lost at post-measurement were compared with the remaining participants on age, gender, education, number of cigarettes smoked per day, nicotine dependence, and intention to quit. In the entire sample, participants lost at post-measurement did not differ significantly from the remaining participants on any of these variables. In the telephone counselling condition, participants lost at post-measurement smoked significantly more cigarettes per day at baseline (*M*=18.8, *SD*=11.3) compared to the remaining participants (*M*=15.4, *SD*=7.5, *t*=1.99, *p*=.05). No other differences were found on the assessed variables.

## Results

### Descriptive statistics at baseline

Table 1 displays sample characteristics at baseline for the entire sample and by condition. At baseline, there were no significant differences between the telephone counselling condition and the self-help brochure condition in the assessed variables.

**Table 1:** Characteristics of participants at baseline

**Characteristics**	**Total sample**	**Proactive telephone counselling**	**Self**-**help brochure**	***p-*value**
	**(N** **=512)**	**(n** **=256)**	**(n** **=256)**	
Age (*M*, *SD*)	42.2 (5.4)	42.3 (5.9)	42.0 (5.1)	.59
Gender (%)				
Female	52.5	51.2	53.9	.54
Nationality (%)				
Dutch	97.9	97.7	98.0	.76
Education (%)				
Low	15.2	16.4	14.1	
Medium	56.6	56.3	57.0	
High	26.2	25.4	27.0	.74
Marital status (%)				
Never married	12.5	12.9	12.1	
Married	67.6	67.6	67.6	
Divorced/separated	19.1	19.1	19.1	
Widowed	0.6	0.4	0.8	.94
Employment status (%)				
Unemployed	15.8	14.5	17.2	
Casual	3.5	3.9	3.1	
Part time	37.5	35.2	39.8	
Full time	43.0	46.5	39.5	.38
Cigarettes per day (*M*, *SD*)	16.2 (7.8)	15.7 (8.0)	16.8 (7.7)	.14
Years of smoking (*M*, *SD*)	24.9 (7.7)	25.1 (7.4)	24.6 (8.0)	.43
FTND score (*M*, *SD*)	4.0 (2.4)	4.0 (2.4)	4.0 (2.4)	.81
Ever made a quit attempt (%)				
Yes	95.3	95.7	94.9	.68
Quit attempt in past 12 months (%)				
Yes	36.1	38.3	34.0	.31
Quitting intention (%)				
Within one month	33.6	33.6	33.6	
Within 6 months	33.0	35.2	30.9	
Within 12 months	23.4	20.3	26.6	
Not within 12 months	9.7	10.9	8.6	.31
Partner smoking (%)				
Yes	33.4	30.9	35.9	.20
Cardiovascular disease				
Yes	1.6	1.2	2.0	.48
Chronic respiratory illness				
Yes	7.8	7.0	8.6	.51
Chronic respiratory illness child (%)				
Yes	14.6	14.5	14.8	.90
Confidence in quitting (0-10)	6.1 (2.0)	6.1 (1.9)	6.1 (2.0)	.82
Importance of quitting (0-10)	8.9 (1.6)	8.9 (1.5)	8.9 (1.6)	.98

Note. *FTND*, Fagerström Test for Nicotine Dependence.

### Reach and costs of mailings distributed through primary schools

Reach of mailings was defined as the ratio of the number of participants enrolled to the number of participants eligible (i.e., recruitment efficiency). In total, approximately 35,000 mailings were distributed to primary schools, which led to the recruitment of 512 smoking parents out of approximately 10,000 households (30%) which are estimated to include at least one smoking parent [37,38], yielding a response rate of approximately 5%.

The total cost for recruitment was 11,131 euro (approximately 14,728 USD), consisting of 2,732 euro in personnel cost (for the principal investigator and a research assistant), 7,467 euro in copy charges (making and mailing the materials), and 300 euro in telephone cost. Overall cost per enrolled participant was 21.74 euro (approximately 28.31 USD).

### Use and acceptability of telephone counselling

Tables 2 and 3 display use and acceptability of telephone counselling. A total of 224 participants (88%) received at least one counselling call, and 212 (83%) received at least three calls. Of participants who received calls, the mean number of calls received was 5.7 (*SD*=1.7).

**Table 2:** **Reported use of telephone counselling and self**-**help brochure at post**-**measurement among the intention**-**to**-**treat population** (**and among recipients**)

	**Telephone counselling condition**		**Self**-**help brochure condition**	
Received call(s)/brochure	Yes	87.5%	Yes	89.1%
	No	2.0%	No	7.0%
Number of calls taken/amount of brochure read	1-2 calls	4.3% (4.9%)	Not read	5.1% (5.7%)
	3-4 calls	15.6% (17.9%)	Read less than half	10.9% (12.3%)
	5-6 calls	32.4% (37.2%)	Read more than half	7.8% (8.8%)
	7 or more calls	34.8% (39.9%)	Read in full	64.8% (73.1%)
Use of tips	None	7.8% (9.0%)	None	37.5% (42.1%)
	A few tips	59.4% (68.2%)	A few tips	45.3% (50.9%)
	A lot of tips	19.9% (22.9%)	A lot of tips	6.3% (7.0%)

**Table 3:** **Evaluation of telephone counselling and self**-**help brochure at post**-**measurement**

			**Recipients**-**only**		**Intention**-**to**-**treat**	
			**Telephone counselling condition**	**Self**-**help brochure condition**	**Telephone counselling condition**	**Self**-**help brochure condition**
			**(n** **=224)**	**(n** **=228)**	**(n** **=256)**	**(n** **=256)**
Telephone counselling brochure helped with	Motivation to quit or stay quit	Not at all	5.4%	22.9%	4.7%	20.3%
		A little	27.8%	63.4%	24.2%	56.3%
		A lot	66.8%	13.7%	58.2%	12.1%
	Withdrawal	Not at all	9.4%	28.6%	8.2%	25.4%
		A little	39.5%	56.8%	34.4%	50.4%
		A lot	51.1%	14.5%	44.5%	12.9%
	Cravings to smoke	Not at all	8.1%	31.3%	7.0%	27.7%
		A little	35.0%	57.3%	30.5%	50.8%
		A lot	57.0%	11.5%	49.6%	10.2%
	Triggers of craving or difficult situations	Not at all	9.0%	25.6%	7.8%	22.7%
		A little	35.9%	65.2%	31.3%	57.8%
		A lot	55.2%	9.3%	48.0%	8.2%
	Preventing a lapse or relapse	Not at all	10.3%	27.8%	9.0%	24.6%
		A little	32.7%	61.7%	28.5%	54.7%
		A lot	57.0%	10.6%	49.6%	9.4%
	Motivation after a lapse or relapse	Not at all	10.3%	27.8%	9.0%	24.6%
		A little	30.5%	58.6%	26.6%	52.0%
		A lot	59.2%	13.7%	51.6%	12.1%
Received	Emotional support	Not at all	22.4%	56.8%	19.5%	50.4%
		A little	34.1%	40.1%	29.7%	35.5%
		A lot	43.5%	3.1%	37.9%	2.7%
	Practical tips	Not at all	5.4%	19.4%	4.7%	17.2%
		A little	18.4%	55.9%	16.0%	49.6%
		A lot	76.2%	24.7%	66.4%	21.9%
Length of telephone counselling/brochure		Too short	10.8%	15.0%	9.4%	13.3%
		About right	84.8%	81.9%	73.8%	72.7%
		Too long	4.5%	3.1%	3.9%	2.7%
Overall satisfaction with telephone counselling/brochure		Unsatisfied	6.7%	22.5%	5.9%	19.9%
		Satisfied	41.7%	72.7%	36.3%	64.5%
		Very satisfied	51.6%	4.8%	44.9%	4.3%

Of all participants randomized to telephone counselling, the majority reported that the calls helped with motivation to quit or stay quit (82%), withdrawal (79%), cravings to smoke (80%), dealing with triggers of craving or difficult situations (79%), preventing a lapse or relapse (78%), or motivation after a lapse or relapse (78%). Also, the majority of participants received emotional support (68%) and practical tips (82%) from the counsellor. Most participants reported that they made use of these tips (79%). The majority of participants (74%) thought that the length of telephone counselling was about right. Overall, 81% were satisfied or very satisfied with telephone counselling and 67% reported that they would make use of telephone counselling again.

Of all participants randomized to telephone counselling, 211 (82%) recalled receiving at least one accompanying booklet. A total of 125 participants (49%) read the booklets in full, and 66 participants (26%) read at least some parts of the booklets. The majority (57%) reported that they have made use of the tips provided in the booklets. The majority of participants reported that the accompanying booklets helped with motivation to quit or stay quit (70%), withdrawal (69%), cravings to smoke (67%), dealing with triggers of craving or difficult situations (67%), preventing a lapse or relapse (66%), or motivation after a lapse or relapse (62%). Overall, 72% were satisfied or very satisfied with the booklets. Results are displayed in Table 4.

**Table 4:** **Evaluation of accompanying booklets in the telephone counselling condition at post**-**measurement among the intention**-**to**-**treat population**


Recalled receipt of booklet(s)		Yes	82.4%
		No	7.0%
Amount read		None	7.8%
		Less than half	10.5%
		More than half	15.2%
		In full	48.8%
Booklet(s) helped with	Motivation to quit or stay quit	Not at all	12.5%
		A little	40.2%
		A lot	29.3%
	Withdrawal	Not at all	13.3%
		A little	40.6%
		A lot	28.1%
	Cravings to smoke	Not at all	14.8%
		A little	41.8%
		A lot	25.4%
	Triggers of craving or difficult situations	Not at all	14.8%
		A little	42.6%
		A lot	24.6%
	Preventing a lapse or relapse	Not at all	16.4%
		A little	43.4%
		A lot	22.2%
	Motivation after a lapse or relapse	Not at all	19.9%
		A little	37.9%
		A lot	24.2%
Use of tips		None	25.0%
		A few tips	52.3%
		A lot of tips	4.7%
Overall satisfaction with booklet(s)		Unsatisfied	10.2%
		Satisfied	57.0%
		Very satisfied	14.8%

### Use and acceptability of self-help brochure

Tables 2 and 3 display use and acceptability of the self-help brochure. Of all participants randomized to the self-help brochure condition, 228 (89%) recalled receiving the self-help brochure. A total of 166 participants (65%) reported that they read the brochure in full, 48 (19%) read at least some parts of the brochure, and 13 participants (5%) did not read the brochure.

Of all participants randomized to the self-help brochure condition, the majority reported that the brochure helped with motivation to quit or stay quit (68%), withdrawal (63%), cravings to smoke (61%), dealing with triggers of craving or difficult situations (66%), preventing a lapse or relapse (64%), or motivation after a lapse or relapse (64%). A total of 38% of participants reported that they received emotional support, 72% received practical tips, and 52% reported that they have made use of these tips. Most participants (73%) thought that the length of brochure was about right. Overall, 69% were satisfied or very satisfied with the brochure.

### Comparison between telephone counselling and self-help brochure

Use and acceptability of telephone counselling were compared to use and acceptability of the self-help brochure. Participants randomized to telephone counselling were significantly more likely to report that cessation support helped with motivation to quit or stay quit (*χ*^*2*^ = .28.32, *p*<.001), withdrawal (*χ*^*2*^ = 26.87, *p*<*.0*01), cravings to smoke (*χ*^*2*^ = 38.18, *p*<.*001*), dealing with triggers of craving or difficult situations (*χ*^*2*^ = 21.57, *p*<.001), preventing a lapse or relapse (*χ*^*2*^ = 22.13, *p*<.001), or motivation after a lapse or relapse (*χ*^*2*^ = 22.13, *p*<.001). Moreover, participants randomized to telephone counselling were significantly more likely to report that they received emotional support (*χ*^*2*^ = 55.59, *p*<.001), they were more likely to receive practical tips (*χ*^*2*^ = 20.24, *p*<.001), and they were more likely to make use of these tips (*χ*^*2*^ = 64.79, *p*<.001). Overall, significantly more participants were satisfied or very satisfied with telephone counselling compared to the self-help brochure (*χ*^*2*^ = 22.27, *p*<.001).

## Discussion

The present study sought to evaluate the feasibility and acceptability of connecting smoking parents to cessation support through their children’s primary schools. As with other populations, recruiting smokers into clinical trials is challenging [39,40]. In the present study, the distribution of 35,000 mailings through primary schools led to the recruitment of 512 smoking parents out of approximately 10,000 households (30%) which are estimated to include at least one smoking parent [37,38], yielding a response rate of approximately 5%, which is in line with earlier studies that require participants to respond to printed information material or mass media [23-26]. It should be noted that the response rate yielded by the present approach is likely to be an underestimation of the response rate which may potentially be achieved using the present approach. First, the present study employed several inclusion criteria (e.g., willingness to fill out questionnaires, participation as parent–child dyad). The response rate is likely to be higher when no inclusion criteria are employed. In line with this, the number of smokers who initially responded to the study invitation letters was considerably higher than the number of smokers who eventually enrolled in the present study (i.e., returned informed consent and baseline questionnaire). Second, the number of eligible subjects in the target population constitutes an estimation which may be subject to imprecision. Overestimation of the prevalence of parental smoking and non-adherence to instructions in schools and children may have lead to an underestimation of the actual response rate among smoking parents. However, previous studies have employed similar procedures (i.e., procedures using estimations of the denominator) to determine rates of recruitment in defined populations [24,26]. It should be noted that, even though the response rate of smoking parents to one-time mailings was rather low, the level of motivation to quit in smoking parents who responded to the mailings was quite diverse (two-thirds of respondents were not ready to quit within one month), providing preliminary evidence that low-intensity outreach targeting both smokers who are not yet ready to quit as well as smokers who are ready to quit may engage smokers with varying characteristics and levels of motivation to quit. In the Netherlands, less than 1% of smokers contact the national quitline [7]. Therefore, even low-intensity outreach (e.g., one-time mailings) may be useful in increasing smoker’s exposure to and use of cessation support. The findings are in line with previous research demonstrating high recruitment efficiency as well as high cost-effectiveness of recruitment strategies which disseminate information material to target audiences through print and media [41]. The fact that half of the approached schools agreed to distribute mailings to parents indicates that schools generally approve of offering cessation support to smoking parents and are willing to participate in school-based smoking cessation promotion programs when demands on schools are kept to a minimum.

The present study offers several directions for future research. First of all, future studies will need to examine factors that influence the response to offers of cessation support in samples of nonvolunteer smokers. Possibly, offering a variety of cessation support services (nicotine replacement therapy, medication, behavioural counselling, web-based support, self-help material) may improve use of cessation support among smokers. Also, periodic mailings may increase the response rate among smokers. In smokers, motivation to quit is unstable over time and may change rather spontaneously [42,43]. Repeated offers of cessation support may capitalize on these variations in smoker’s motivation to quit. To achieve an impact on smoking parents at the population level, proactive outreach efforts may additionally capitalize on ‘teachable moments’ in clinical settings such as consultancy and hospitalization for respiratory illness in children, prenatal consultancy, or postpartum hospital stays [17,22].

While recruitment of smokers into cessation support remains challenging, the reported use of cessation support among proactively recruited smokers was high and evaluations of cessation support were remarkably positive. Among all participants randomized to telephone counselling, almost 90% accepted at least one counselling call and more than 80% received three or more counselling calls. Overall, more than 80% of smoking parents were satisfied or very satisfied with telephone counselling. There was very little variability in the evaluation of telephone counselling, indicating that telephone counselling was generally well-received among smoking parents. In addition to telephone counselling, smoking parents also received three accompanying booklets. The accompanying booklets were read by 75% of parents and 72% were satisfied with the booklets. Supplementary materials may provide tailored information to target audiences and may be used as an increment or booster to generic interventions (e.g., telephone counselling), as they were well-received and read by participants who participated in telephone counselling. With regard to the self-help brochure, more than 80% reported that they read at least some parts of the brochure and nearly 70% were satisfied or very satisfied with the brochure. Self-help materials are a cost-effective method to support otherwise unaided quit attempts, which can be disseminated easily. In general, self-help material seems to be well-received and may be of interest to smoking parents, though findings clearly demonstrate that smoking parents are more favourable about telephone counselling than self-help material. Interpersonal contact and the counsellor’s use of motivation interviewing techniques (e.g., empathic listening, non-judgemental exploration of ambivalence) may be one reason for the positive evaluation of telephone counselling among smoking parents. The present findings are in line with previous studies showing that quitline services are well-received, even by non-volunteer smokers [7,28].

Several limitations should be acknowledged. First of all, the results of the present study are based on self-report. Social desirability or memory biases may have influenced the recall of the use of cessation support. Also, attrition was significantly higher in the telephone counselling condition compared to the self-help brochure condition, indicating selective drop-out (possibly due to differences in contact frequency or differences in satisfaction with treatment). Yet, the attrition rate in the present study was rather low, and few differences were observed between the remaining participants and participants lost to attrition. Also, all results pertain to the intention-to-treat population, therefore satisfaction with treatment is likely to be underestimated rather than overestimated. As study participants were aware of the two-arm design, it is possible that treatment preferences at the start of the study may have affected treatment evaluations, possibly resulting in an underestimation of satisfaction with treatment, particularly among participants receiving the self-help brochure. It should be acknowledged that study procedures that deviated from standard practice procedures may limit generalizability (completion of assessments and use of incentives). The effectiveness of the proactive telephone counselling offered to smoking parents will be examined in a separate manuscript once follow-up data collection has been completed.

## Conclusions

To summarize, the present study evaluated use and acceptability of telephone counselling and self-help material among smoking parents who were recruited into cessation support using mailings distributed through primary schools. In the present study, the response rate to offers of cessation support was rather low (5%), though it may be improved by offering varying types of cessation services and employing fewer requirements for participation. Once recruited into cessation support, both telephone counselling and self-help material were well-used and well-evaluated by smoking parents. The findings demonstrate that parents were clearly more positive about telephone counselling compared to self-help materials.

## Endnote

^a^ Dutch name of brochure: *Stoppen met roken*: *Willen en kunnen*.

## Competing interests

The authors declare that they have no competing interests.

## Authors' contributions

KS is responsible for the data collection, data analysis, and report of study results. RO, MK, JB, and RE are supervisors and grant applicators. All authors read and approved the final manuscript.

## Pre-publication history

The pre-publication history for this paper can be accessed here:

http://www.biomedcentral.com/1471-2458/13/381/prepub
